# Analysis of the Leaves and Cones of Lithuanian Hops (*Humulus lupulus* L.) Varieties by Chromatographic and Spectrophotometric Methods

**DOI:** 10.3390/molecules27092705

**Published:** 2022-04-22

**Authors:** Žydrūnas Stanius, Mantas Dūdėnas, Vilma Kaškonienė, Mantas Stankevičius, Elżbieta Skrzydlewska, Tomas Drevinskas, Ona Ragažinskienė, Kęstutis Obelevičius, Audrius Maruška

**Affiliations:** 1 Instrumental Analysis Open Access Centre, Faculty of Natural Sciences, Vytautas Magnus University, Vileikos St. 8, LT-44404 Kaunas, Lithuania; zydrunas@armgate.lt (Ž.S.); mantas.dudenas@alumni.vdu.lt (M.D.); vilma.kaskoniene@vdu.lt (V.K.); mantas.stankevicius@vdu.lt (M.S.); tomas.drevinskas@vdu.lt (T.D.); 2 Department of Analytical Chemistry, Medical University of Bialystok, Mickiewicza St. 2D, 15-222 Białystok, Poland; elzbieta.skrzydlewska@umb.edu.pl; 3 Sector of Medicinal Plants, Botanical Garden of Vytautas Magnus University, Ž. E. Žilibero St. 6, LT-46324 Kaunas, Lithuania; ona.ragazinskiene@vdu.lt (O.R.); kestutis.obelevicius@vdu.lt (K.O.)

**Keywords:** xanthohumol, QUENCHER, total phenolic compounds content, radical scavenging activity, supercritical fluid extraction

## Abstract

This work involves a comprehensive chemical composition analysis of leaf and cone samples of Lithuanian hop varieties. This study aimed to determine the chemometric properties of the leaves and cones of five Lithuanian hop varieties. Determined properties were the following: (a) xanthohumol content, (b) phenolic compounds, (c) flavonoids, (d) radical scavenging activity, and (e) the qualitative composition of volatile compounds. The total content of phenolic compounds in aqueous 75% methanolic extracts varied between 31.4–78.2 mg of rutin equivalents (RE)/g, and the concentration of flavonoids was between 11.0–23.3 mg RE/g. Radical scavenging activity varied between 34.4–87.2 mg RE/g. A QUENCHER analysis procedure showed 91.7–168.5 mg RE/g of the total phenolic compound content, 12.7–21.4 mg RE/g of flavonoids, and 48.4–121.0 mg RE/g of radical scavenging activity. ‘Fredos taurieji’ and ‘Fredos derlingieji’ varieties have shown maximum values of phenolic compounds and radical scavenging activity both in leaf and cone suspensions. These varieties accumulated a higher amount of xanthohumol in leaves. The concentration of xanthohumol in the samples varied between 0.0014–0.2136% of dry mass, with the highest concentration in the cones of ‘Kauno gražieji’. We identified 19 volatile compounds in leaves, and in cones, we identified 32. In both of them, α-humulene and β caryophyllene dominated. ‘Raudoniai’ leaves were exceptional in their aroma due to dominating compound nagina ketone (Kovats index 1306). The QUENCHER procedure has shown a great potential for the unextractable residue of hop raw material. Further investigation and valorization of different hop biomass components, not only cones, are essential.

## 1. Introduction

Hop (*Humulus lupulus* L.) is a dioecious perennial plant that belongs to the *Cannabaceae* family. Hops have been known for over 2000 years and are widely recognized as one of four essential ingredients used in brewing, infusing beer with bitterness and specific aromas. To Lithuania, hops were introduced in the Middle Ages by the Teutonic Order and the Livonian Order from Koenigsberg and Riga. Many wild forms are still found naturally growing and in the gardens. Hops are also acknowledged as a herbal plant in traditional medicine, purposed for the treatment of sleeplessness, stimulating gastric secretion, and having anti-inflammatory and antimicrobial effects [1]. Some scientific studies were done assessing traditionally anticipated biologically active compounds in hops, which show their antioxidant [1], estrogenic [2], and antimicrobial [3,4] activities. Polyphenols are a crucial part of hop antioxidant activity [5], being effective in many different chemical oxidation systems [1]. The highest reported phyto-estrogenic properties belong to 8-prenylnaringenin (8-PN) mostly found in the strobili of hops [6], and there are a number of commercial dietary supplements containing standardized 8-PN to help relieve menopausal symptoms [7]. Hops also possess antibacterial compounds, of which bitter β-acids are the most active [1]. The antibacterial effect of bitter β-acids is based on damaging the cytoplasmic membrane of the bacteria, interfering with the active transport of sugars and amino acids, and lowering intracellular pH [8].

One of the reasons hops gained new intense emphasis in medicinal studies is their anti-cancer activity [9]. Hop’s most abundant prenylated flavonoid is xanthohumol. It has anti-cancer properties against colon cancer [6], pancreatic cancer [9], larynx cancer [10], and many other cancer forms [11]. There are several pathways to how xanthohumol enacts its anti-cancer activity mechanism. This compound participates in chemo-preventive, anti-angiogenic, anti-invasion, anti-inflammatory, proapoptosis activities, and modulation of autophagy [12]. The research conducted by Wang et al. [13] has shown that xanthohumol can also inhibit HIV-1 replication in peripheral blood mononuclear cells.

Xanthohumol and other biologically active hop compounds used in scientific research are predominantly extracted from hop strobiles. Less attention is given to other hop plant material, such as the leaves, which constitutes a relatively significant part of plant biomass. There is a lack of literature data on hop leaves analysis, especially about the abundance of xanthohumol and volatile compounds in leaves. 

Traditional solvent extraction [14,15,16], accelerated solvent extraction [17], solid phase extraction [18], solid phase microextraction [17,19], supercritical fluid extraction [17,20], steam distillation [17,21], and other extraction methods are used for the extraction of biologically active compounds from hop plant material. Different extraction methods can vary by specificity and extraction potential for different analytes. The QUENCHER procedure is an important technique that involves a colorimetric reaction with a whole sample suspension [22]. This method provides integral results of a certain activity’s compounds that belong to the (a) dissolved and (b) solid parts. Comparing results of the QUENCHER procedure and results of conventional liquid extraction provides valuable information on the additional potential of raw material. The content of biologically active compounds depends on several factors: a plant’s variety [4], its vegetation phase [23], climatic conditions [24], or even the chemotype or ecotype of the plant [25]. Therefore, it is important to have a descriptive analysis of different hop varieties. 

This study aimed to: (a) determine the content of xanthohumol, (b) screen the total content of phenolic compounds, flavonoids, and radical scavenging activity, and (c) evaluate the qualitative composition of the volatile compounds of the leaves and cones of five Lithuanian hop varieties. These varieties were nurtured by Dr. Stasys Gudanavičius between 1952–1975 using the hybridization method and grown in the Botanical Garden of Vytautas Magnus University. Nurtured plants have adopted local edaphoclimatic conditions, high productivity, and resistance to plant diseases. To our knowledge, it is the first study on the evaluation of xanthohumol content in the leaves and cones of Lithuanian hop varieties. The QUENCHER approach was applied for hop analysis for the first time as well.

## 2. Results and Discussions

A comparative study of the composition of biologically active substances has been performed. The bio-activity in hop cones and hop leaves of the up-to-date nurtured five Lithuanian hop breeds was analyzed. Determined chemometric parameters are the following: (a) the total content of phenolic compounds, (b) the flavonoid compounds, (c) the radical scavenging activity, (d) the qualitative composition of volatile compounds, and (e) the total content of xanthohumol in the leaves and cones of Lithuanian hop varieties. Two different procedures for the determination of extractable and biologically active compounds in the suspension were used. These two procedures helped to compare data of conventional solvent extraction and whole raw material.

### 2.1. Total Content of Phenolic Compounds, Flavonoids, and Radical Scavenging Activity

Initially, the total content of phenolic compounds in the samples was analyzed. After solvent extraction, the concentration of phenolic compounds varied between 31.4–63.6 mg RE/g in the leaves and from 71.7 to 78.2 mg RE/g in the cones of analyzed varieties. The highest concentration of polyphenols was determined in the extracts of the leaves of ‘Fredos derlingieji’ and the extracts of the cones of ‘Fredos taurieji’. 

The QUENCHER, which stands for QUick, Easy, New, CHEap, and Reproducible, is a time and cost-saving methodology that does not need the extraction of analytes of interest. It therefore proposes the measurement of antioxidant capacity of both soluble and insoluble compounds. This method is based on the surface reaction between solid (bound or trapped antioxidants) and liquid (soluble free radicals) phases. This method has a critical point of grinding and weighing, which affects measurements’accuracy [26], which was addressed in this work. The concentration of polyphenols using the QUENCHER procedure varied between 111.4–168.5 mg RE/g in the leaves and between 91.7–134.8 mg RE/g in the cones of hops. The highest content of phenolic compounds was determined in the raw materials of the leaves of ‘Fredos taurieji’ and the cones of ‘Fredos derlingieji’. As shown in Table 1, the total content of phenolic compounds using the QUENCHER procedure is 1.2–3.5 times higher than using conventional solvent extraction for 24 h. A big difference of the estimations obtained by the QUENCHER procedure and the soluble part of the samples shows the importance of the evaluation of the whole raw material. It also points out a gap existing in the evaluation of plant samples and valorization of plant raw material, since only substances soluble in extracts are commonly estimated.

Such a difference is due to the presence of insoluble (in aqueous 75% methanol mixture) phenolic compounds used for conventional extraction. In contrast, the QUENCHER procedure allows assessing of the full suspension, i.e., both soluble and insoluble analytes. In addition, in the QUENCHER procedure, the total content of phenolic compounds in the leaf samples was predominantly higher than it was in the same variety of cone samples. Such an outcome was the opposite of the results obtained with solvent extraction for most investigated varieties. The difference in results may be explained by the proposition that leaves may have more insoluble phenolic compounds in aqueous 75% methanol; therefore, they cannot be extracted efficiently. Another reason could be that some measurable compounds are trapped or encapsulated in the plant matrix and cannot physically pass to the extraction solvent. In contrast, cones have some external resins that solvent extracts more easily.

Furthermore, it was calculated that the total content of phenolic compounds differs significantly (*p* ≤ 0.05) between QUENCHER and conventional solvent extraction for all leaf samples.

Different authors use different approaches to represent the results (for example, using chlorogenic acid equivalents). Abram et al. [3] analyzed the total content of polyphenols in hop leaves and cones samples. They found that the polyphenol content in solvent extracts of ‘Aurora’ cones, harvested between 2008–2010, was 2.7–4.6 times higher than the leaves. Our study revealed that the total content of phenolic compounds in the cone samples of analyzed varieties was 1.1–2.4 times higher than leaf samples. 

The total content of flavonoids measured is remarkably lower than the total content of phenolic compounds. It is because flavonoids represent only one group of phenolic compounds. As it is seen in Table 1, there is no significant difference in flavonoid content between the samples of leaves and cones using solvent extraction and the QUENCHER procedure for the majority of samples. A small difference between the results obtained by the solvent extraction and the QUENCHER procedure suggests that most flavonoids are soluble in aqueous methanol and were efficiently extracted using the conventional aqueous 75% organic solvent extraction. The total content of flavonoids in solvent extracts of leaves varied between 11.0–18.1 mg RE/g, whereas in suspended material measurements, concentrations varied between 12.9–21.4 mg RE/g. The highest concentration of flavonoids in the samples of leaves was in the solvent extract of ‘Fredos taurieji’ and the suspension of ‘Raudoniai’. Solvent extracts of hop cones contained between 14.4–23.3 mg RE/g of flavonoids. Similar values of 12.7–20.2 mg RE/g were obtained, assessing the whole suspension using the QUENCHER procedure. The variety ‘Kauno gražieji’ has shown the highest concentration of flavonoids in both conventional extraction and the QUENCHER procedure results.

Krofta et al. [5] applied high-performance liquid chromatography, based on Analytica EBC methods, to determine hop flavonoids. The study revealed that the extracts of dried cone samples of ‘Premiant’ and ‘Sladek’ varieties contained 9.6 and 15.5 mg/mL of flavonoids, respectively. Even though hop cones are well researched, there is a lack of detailed analysis of the phytochemical composition of hop leaves. There is no data published to the authors’ knowledge, including flavonoid analysis in the hop leaves. 

Radical scavenging activity of the samples was assessed using a DPPH radical bleaching (measured at 517 nm) colorimetric method. As seen in Table 1, radical scavenging activity using the QUENCHER procedure resulted in higher values in most cases. Since the total content of phenolic compounds was 1.2–3.5 times higher using QUENCHER than the solvent extract, radical scavenging activity has not shown the same trend here. It may be determined by phenolic compounds, insoluble in aqueous 75% methanol, and do not possess any radical scavenging activity in the DPPH system. Radical scavenging activity in the solvent extracts of leaves varied between 34.4–69.5 mg RE/g and 65.1–87.2 mg RE/g for cone extracts. Radical scavenging activity in the suspension of the raw materials varied from 48.4 to 121.0 mg RE/g for leaves and between 66.6–102.0 mg RE/g for cones. There was a significant difference (*p* ≤ 0.05) of radical scavenging activity between solvent extraction and the QUENCHER procedure for all leaf samples. At the same time, there were no significant differences in radical scavenging activity between two out of five analyzed samples of cones. There was a strong correlation between the total content of phenolic compounds and radical scavenging activity in aqueous 75% methanol extracts (r = 0.93), supporting the idea that polyphenols play an essential role in the antioxidant activity of hops [5].

It’s also problematic to parallel results of radical scavenging activity with other studies as there is no established unified system for expressing results. Abram et al. [3] analyzed the antioxidant activity of hop leaves and cones. The study revealed that solvent extracts of hop leaves have a radical scavenging activity of 3 mg/g of dry weight. The study by Krofta et al. [5] has shown that in the DPPH radical system, the antioxidant activity of cones of ‘Premiant’ and ‘Agnus’ varieties were 59.6% and 49.0%, respectively. Palombini et al. [26] analyzed the antioxidant capacity of Brazilian rice cultivars using the QUENCHER procedure. The analysis showed that the antioxidant activity of ‘BRS Primavera’ cultivar rice in the DPPH system was 794.51 µmol TEAC/g (TEAC—Trolox equivalent antioxidant capacity). Such a multitude of measurement result dimensions does not help compare measured values and creates a need for conversion algorithms or a unified expression system. To harmonize spectrophotometric tests of total polyphenols, flavonoids, and radical scavenging activity, we use the same calibration standard compound, i.e., rutin, polyphenol flavonoid, and DPPH radical scavenger at the same time. 

### 2.2. Qualitative Composition of Volatile Compounds

Supercritical carbon dioxide extraction was used to study the qualitative and quantitative composition of volatile compounds in the hop cones and leaves. As it could be surmised, hop cones are richer than leaves in essential oil compounds. Analyses showed 34 substances, of which 32 were identified. In leaves, 19 compounds were identified. Results of the gas chromatography analysis are given in Table 2.

It is important to note that identified compounds in less than four samples were omitted from further data analysis. Major hop cone volatile compounds were identified as: β-caryophyllene, α humulene, β-pinene, sylvestrene, β-cubebene, β-selinene, α-muurolene, and caryophyllene oxide. Borneol, estragole, and copaene were identified in the leaf samples, as well as compounds that are characteristic and found in cone samples, such as α-bergamotene, β-farnesene, γ muurolene, and γ and δ-cadinene, etc. As evident from Table 2, the predominant compounds in all the samples were β-caryophyllene and α-humulene. These compounds are characteristic volatile compounds of hops [2,21]. The cones of hops can be distinguished by a far wider variety of volatile compounds than leaves. Quantities of volatile compounds in cones are significantly higher than in leaves as well. Along with shared compounds, the predominant volatile compound of cones, β-myrcene, was not identified in any leaf sample. When comparing the percentage composition of β-farnesene between leaves and cones, quantitative differences are essential. As seen from Table 2, β-farnesene is a predominant compound in cone samples; however, it takes just a fraction of the composition of volatile compounds in leaves. In addition, a minor compound in all samples, nagina ketone (Kovats index 1306), was the most dominant in leaf samples of ‘Raudoniai’, making 78.70% of all volatile compounds. It makes ‘Raudoniai’ an extraordinary smell variety, which is similar to the aroma of the black currant leaves. The reasons for such results are unknown and might require further studies.

**Figure 1:**
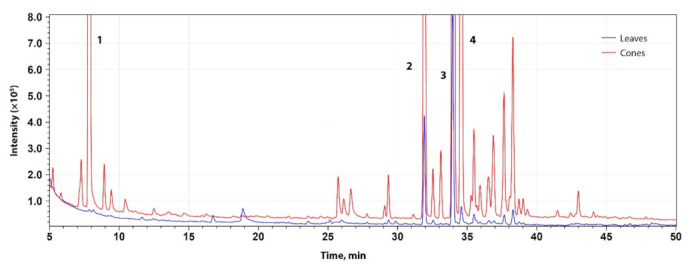
Gas chromatogram and characteristic volatile compounds of supercritical fluid extracts of leaves and cones of ‘Fredos derlingieji’ (1—β-myrcene; 2—β-caryophyllene; 3—α-humulene; 4—β-farnesene).

The chromatographic profile perfectly demonstrates that the quantity of volatiles was a few times lower in the leaves than cones, as shown in Figure 1. 

In our previous study [21], clustering analysis of different hop varieties and wild forms based on hop essential oil composition was carried out to classify hops according to their chemometric similarity. It was concluded that the main volatile compounds of hop samples analyzed were β-myrcene, α-humulene, β-farnesene, caryophyllene oxide, and humulene epoxide II. When comparing differences in composition between cone samples of the same variety, we have earlier shown predominant compounds are the same; however, there are differences in qualitative and quantitative compositions resulting in different extraction methods and the harvest year. In another study [17] we showed that β-caryophyllene, γ-muurolene, γ-cadinene, and other compounds can be extracted using solid-phase microextraction. Whereas supercritical fluid extraction allowed to additionally extract borneol, β-cubebene, α-bergamotene, β-selinene, α-farnesene, eremophylene, and other compounds. Another study has shown that ylangene, copaene, 2-nonanone, 2-decanone, and other compounds were also present in supercritical carbon dioxide extract of ‘Marynka’ variety cones [20].

### 2.3. Xanthohumol Content and Variation of Bitter Acids 

**Figure 2:**
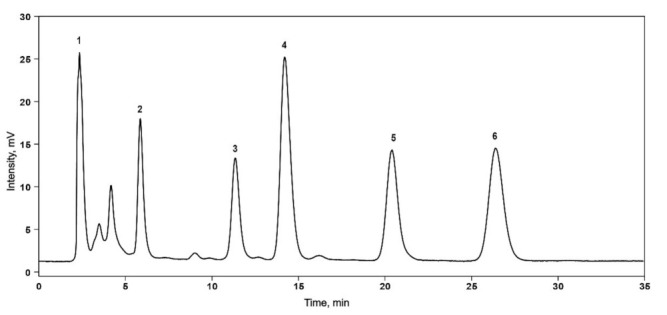
HPLC chromatogram of aqueous 75% methanol extract of cones of ‘Kauno gražieji’ hop variety. (1—compounds that are not retained by reversed phase stationary phase; 2—xanthohumol; 3—cohumulone; 4—humulone; 5—colupulone; 6—lupulone; λ = 348 nm).

Ceh et al. [16] predicted that the leaves of hops should contain a small amount of xanthohumol. Our research addressed this prediction by including leaf samples in xanthohumol analysis by the HPLC method. A representing chromatogram is given in Figure 2.

Compound content was assessed using a calibration curve in the concentration range of 0.03–100 µg/mL. The xanthohumol content in the leaves and cones of Lithuanian hop varieties is presented in Table 3. 

Leaves contained a small fraction of xanthohumol found in the cones, with the highest concentration in the leaves of ‘Fredos taurieji’, while the lowest concentration was in the leaves of ‘Raudoniai’. Such results may be explained by the fact that xanthohumol is synthesized in lupulin glands that are only found in the cones of hops [1,2]. However, very small amounts of this compound may be present because xanthohumol may be secreted as a part of the resins on the inner side of leaf trichomes [27].

Ceh et al. [16] analyzed xanthohumol concentration in the leaves of multiple hop varieties in different water supply conditions. They investigated the influence of drought stress on xanthohumol concentration using pot and field groups of samples. The analysis has shown that xanthohumol content in the leaves of different hop varieties in the water-pot group varied between 0.003 and 0.031% of dry weight. The highest and the lowest concentrations of xanthohumol were in the leaves of ‘Taurus’ and ‘AH Jug2’ varieties, respectively. According to this study, the concentration of xanthohumol depends more on cultivar than on growing conditions.

Xanthohumol content in the cones of the analyzed varieties varied between 0.1181–0.2136% of dry weight. The results of the analysis are presented in Table 3. There was a medium and strong correlation between xanthohumol and flavonoid content (correlation coefficient r = 0.73), bitter α-acids (r = 0.94), and bitter β-acids (r = 0.80) in the samples of the cones. The overall activity of the lupulin glands may influence such relations because xanthohumol and bitter acids are synthesized in these glands [1,2].

**Figure 3:**
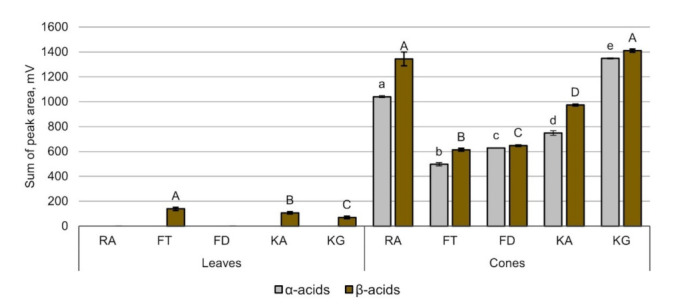
The concentration of α- and β- bitter acids in the samples, expressed as a sum of corresponding HPLC/UV peak area (sample codes see in Table 4. Results represent averaged values of three repetitions. a–e—different letters indicate significant differences between β-acids content among varieties (*p*≤ 0.05); A–D—different capital letters indicate significant differences between α-acids content among varieties (*p* ≤ 0.05)).

As reported by Kac and Vovk [15], the proposed chromatographic method allows the determination of bitter α- and β- acids and their homologues. Along with xanthohumol, bitter α- and β-acids were also identified. It is evident from Figure 3 that aqueous 75% methanol extracts of the leaves of some analyzed varieties contain minimal amounts of bitter β-acids colupulone, and lupulone. Still, there is no α-acids present in the samples of leaves. 

Kac and Vovk [15] analyzed xanthohumol content in the cones of different varieties. Their analysis has shown that xanthohumol concentration in the cones of ‘Savinjski Golding’, ‘Magnum’, and ‘Aurora’ varieties were 0.177%, 0.182%, and 0.288% of dry mass, respectively. Ceh et al. [16] reported that xanthohumol content in ‘Celeia’ and ‘Taurus’ cones were 0.3% and 0.8% of dry mass, respectively. The annual generation of the dry weight of the above-ground part of hops depends on the variety in edaphoclimatic conditions and can reach a maximum cumulative dry mass of 2 tons per acre [28]. Hop cones constitute ca., 30%, and a considerable part of biomass is accumulated in the leaves and vines, which are used as biofertilizers in most cases. Therefore, further research on bioactive compounds, their accumulation in different parts, and their possible applications, such as the valorization of hop plant biomass, particularly of leaves, is vital. 

## 3. Materials and Methods

### 3.1. Plant Material

The cones and leaves were collected from five different Lithuanian *Humulus lupulus* L. varieties: ‘Raudoniai’, ‘Fredos taurieji’, ‘Fredos derlingieji’, ‘Kauno ankstyvieji’, and ‘Kauno gražieji’ growing in the Botanical Garden of Vytautas Magnus University, Kaunas, Lithuania. The same harvest year samples of leaves were collected in July during the emergence of inflorescences of the plant, while the samples of cones were collected in September when the cones were fully mature. Samples were dried and kept in a cool, dry, and dark environment. Before extraction, hop cones and leaves were pulverized with a coffee mill and the fraction below 0.5 mm was obtained. Sample codes and additional information is presented in Table 4.

### 3.2. Chemicals

Alkane C_8_-C_20_ analytical standard (40 mg/L), 2,2-diphenyl-picrylhydrazyl (DPPH), methanol (HPLC grade), rutin (95%), and trifluoroacetic acid (99.9%) were purchased from Sigma-Aldrich (St. Louis, MO, USA); acetonitrile (>99%) and hexametylentetramine (99%) were supplied by Carl Roth (Karlsruhe, Germany); acetic acid (glacial) and aluminum chloride (99.17%) were purchased from Chempur (Piekary Śląskie, Poland); Folin-Ciocalteu phenol reagent and n-heptane (HPLC grade) were purchased from Merck (Darmstadt, Germany); sodium acetate (98%) and sodium carbonate (99.5%) were supplied by Reachem (Petržalka, Slovakia); carbon dioxide (99.9%) and helium (99.999%) gases were purchased from AGA (Vilnius, Lithuania); microcrystalline cellulose was purchased from Chempol (Židlochovice, Czech Republic); and the xanthohumol analytical standard (≥98%) was kindly supplied by Axen Bio (Gdańsk, Poland).

### 3.3. Assessment of Sample Moisture

The samples’ moisture was assessed using a PMB 53 moisture analyzer (Adam Equipment, Oxford, CT, USA). For moisture content, a measurement ≥ 1 g of the sample was placed in the analyzer. All concentrations were recalculated to express values in the dry mass of the samples.

### 3.4. Preparation of Solvent Extracts

Air-dried samples were ground using a coffee mill. Ground sample (0.5 g) was extracted with 20 mL of 75% (vol.) aqueous 75% methanol under continuous shaking for 24 h in an orbital shaker (J&M Scientific, Annandale, MN, USA). Extracts were filtered using a paper filter prior to analysis. 

### 3.5. Spectrophotometric Analysis of Liquid Extracts

The samples’ total amount of phenolic compounds, flavonoids, and radical scavenging activity were analyzed using the Folin–Ciocalteu method, colorimetric aluminum chloride, and 2,2-diphenyl-1-picrylhydrazyl (DPPH) radical methods, respectively, as described in our earlier work [29]. UV/VIS absorbance was measured using Spectronic 1201 (Milton Roy, Ivyland, PA, USA) spectrophotometer. Quantitative calibration was performed using rutin in the range of 0.01–1.00 mg/mL for flavonoids and phenolic compounds, whereas the range of 0.05–0.25 mg/mL was used for radical scavenging activity. Results were expressed as milligrams of rutin equivalents (RE) per one gram of dry sample. All measurement results were expressed as average from three subsequent repetitions. 

### 3.6. QUENCHER Procedure of the Suspended Material

The sum amount of phenolic compounds in whole suspension, i.e., liquid and solid fractions, was evaluated using the QUENCHER procedure. Air-dried samples were pulverized using a coffee mill. Pulverized samples were vigorously shaken to improve homogeneity. Microcrystalline cellulose as an inert material was added at the ratio of 1:9 (sample: cellulose). The mixture was once again vigorously shaken to ensure homogeneity. Ten mg of plant and cellulose mixture was suspended in solution, which consisted of 6000 µL of 4% sodium carbonate, 200 µL of Folin–Ciocalteu reagent, and 80 µL of 75% (vol.) aqueous 75% methanol. The final suspension was incubated in an orbital shaker VWR Mini Shaker (J&M Scientific, Annandale, MN, USA) at ambient temperature for an hour. Solid and liquid phases were separated by centrifugation, and the absorbance of the supernatant was measured at the wavelength of 760 nm. Obtained values were compared against the calibration curve of the rutin standard, and results were expressed as rutin equivalents per gram of the sample. All measurement results were expressed as an average from three subsequent repetitions. 

For the QUENCHER procedure to evaluate the total content of flavonoids in the samples, fifteen milligrams of the sample (plant and cellulose mixture) were suspended in the solution, which consisted of 5760 µL of stock solution (prepared accordingly to our earlier study [17] and 120 µL of 75% (vol.) aqueous 75% methanol. The mixture was shaken in an orbital shaker at room temperature for an hour. Solid and liquid phases were separated by centrifugation, and the absorbance of the supernatant was measured at the wavelength of 407 nm. Final results for total flavonoid amount were calculated using the same methodology as described for flavonoid total amount in liquid extracts. 

QUENCHER procedure was also applied for measuring the radical scavenging activity of solid samples. Five milligrams of the sample were suspended in the solution, which consisted of 18,000 µL of DPPH solution and 240 µL of 75% (vol.) aqueous 75% methanol. The mixture was shaken in an orbital shaker at room temperature in the dark for an hour. The mixture was centrifuged, and the absorbance of the supernatant was measured at the wavelength of 517 nm. The further data processing was done in an analogical way as radical scavenging activity of extracts.

### 3.7. Preparation of Supercritical CO_2_ Extracts

The supercritical fluid extraction was performed using a HP 7680T (Hewlett Packard, Palo Alto, CA, USA) system. Ground sample (0.5 g) was extracted using supercritical CO_2_ at the following conditions: extraction pressure 91 bar; extraction vessel volume 10 mL; 1 mL/min fluid flow rate; and extraction chamber temperature 50 °C (CO_2_ density 0.30 g/mL). Static and dynamic extraction was performed for 2 and 15 min, respectively. The collection of essential oils was carried out on an octadecylsilica (ODS) trap (1 mL) at 10 °C. Elution was performed using 0.7 mL of n-heptane as a desorption solvent at 0.7 mL/min at 45 °C. The extract was analyzed using gas chromatography.

### 3.8. Gas Chromatography-Mass Spectrometry

The analysis of volatile compounds of hop leaves and cones samples after supercritical fluid extraction was performed using GC-2010 (Shimadzu, Kyoto, Japan) gas chromatography system with AOC-5000 (Shimadzu, Kyoto, Japan) autoinjector and GCMS-QP2010 mass-spectrometry detector (Shimadzu, Kyoto, Japan). Separation was carried out using Rtx-5MS (Restek, Bellefonte, PA, USA) column (30 m × 0.25 mm × 0.25 µm) and helium as the carrier gas. The analysis was performed under the following conditions: injector temperature 240 °C; split ratio 1:10; injection volume 1 µL; and flow rate 1.2 mL/min. The column oven temperature was programmed from 60 °C (held for 3 min), heated up to 150 °C at the speed of 2 °C/min, held at 150 °C for 5 min and then heated to 285 °C at the speed of 10 °C/min (held for 8 min). Mass spectrometry detection was performed using electron impact (EI) ionization using 200 °C ion source temperature, 70 eV ionization energy, and 30–400 *m*/*z* scan range. Volatile compounds were identified using NIST05 (NIST, Gaithersburg, MD, USA) mass spectra library and by calculating RI (retention indices), which were calculated using n-alkanes (C_8_–C_20_) retention times for the same analysis conditions as for the samples.

### 3.9. High-Performance Liquid Chromatography

HPLC analysis was carried out using a modular system that consisted of solvent reservoirs, mobile phase pump 9012 (Varian, Palo Alto, CA, USA), mobile phase mixer SP8500 (Spectra-Physics, Milpitas, CA, USA), Cheminert C1 injector (Valco Instruments, Houston, TX, USA), UV detector Linear 206 PHD (Linear Instruments, Shefford, UK), Lichrospher 100 RP-18e 5 × 4.0 mm, 5 µm reversed-phase pre-column and column 125 × 4.0 mm, 5µm (Merck, Darmstadt, Germany). Data was acquired using Clarity Lite Data Acquisition software (Data Apex, Prague, Czech Republic). Xanthohumol concentration in the methanolic extracts was analyzed according to Kac and Vovk [15] method with slight modification. The analysis was carried out under isocratic conditions using solvents A (0.05% trifluoroacetic acid solution in H_2_O) and B (0.05% trifluoroacetic acid solution in HPLC grade methanol) at the ratio of 22:78 (A:B). An injection volume of 20 µL, mobile phase flow rate of 0.7 mL/min, and detection wavelength of 348 nm was used. The peak of xanthohumol was identified using the retention time of xanthohumol analytical standard. 

### 3.10. Statistical Analysis

All measurements were repeated three times, and the results were expressed as an average. The results were processed using Microsoft Excel 2016 (Microsoft, Redmond, WA, USA) software. Average values, standard deviations, relative standard deviations, correlation coefficients, and single-factor ANOVA analysis were carried out using SPSS Statistics 17 (IBM, Armonk, NY, USA) software. A one-way analysis of variance (ANOVA) was used to calculate statistically significant differences of the total content of phenolic compounds, flavonoids, and radical scavenging activity between conventional aqueous 75% methanol extracts and dried raw material of leaves and cones of hops determined by the QUENCHER procedure.

## 4. Conclusions

Adetailed phytochemical composition analysis of the leaves and cones of five Lithuanian hop varieties was carried out. The findings are the following:

(a) The QUENCHER procedure shows 1.2–3.5 times more total content of phenolic compounds than the classical method with conventional solvent extraction.
(b) The QUENCHER procedure reveals matrix encapsulated/adsorbed/insoluble substances.
(c) The leaves of the hops contain phenolic compounds that are insoluble in aqueous 75% methanol.
(d) The content of phenolic compounds and antioxidant activity correlates.
(e) The highest value of phenolic compounds and radical scavenging activity was in the suspensions of leaves and cones of Fredos taurieji’ and ‘Fredos derlingieji’.
(f) The highest concentration of xanthohumol in the leaves was 0.0080%, and in the cones was 0.2136% of dry mass.
(g) The xanthohumol content in hop cones strongly correlates with flavonoid, bitter α-acids, and β-acids contents.
(h) The highest amounts of hop’s bitter acids were determined in ‘Kauno gražieji’ and ‘Raudoniai’.

‘Raudoniai’ leaves were exceptional in their aroma. The study shows that further investigation and valorization of different hop biomass components is of importance, especially the potential of leaves and wines.

## Figures and Tables

**Table 1:** The total content of phenolic compounds, flavonoids, and radical scavenging activity of leaves and cones of analyzed varieties using aqueous 75% methanol (75% (vol.)) extraction and QUENCHER procedure (*n* = 3).

Variety	Total Phenolic Compounds (mg RE/g)		Total Flavonoids (mg RE/g)		Radical Scavenging Activity (mg RE/g)	
	Extract	QUENCHER	Extract	QUENCHER	Extract	QUENCHER
**Leaves**						
RA	53.00 ± 0.44 ^a^	128.35 ± 3.14 ^e^	15.92 ± 0.61 ^a^	21.41 ± 1.97 ^f^	39.77 ± 0.69 ^a^	52.69 ± 5.38 ^d^
FT	60.46 ± 2.43 ^b^	168.46 ± 9.44 ^f^	18.08 ± 0.40 ^b^	18.76 ± 1.08 ^bcf^	67.83 ± 0.78 ^b^	121.02 ± 12.09 ^e^
FD	63.59 ± 1.59 ^b^	164.18 ± 15.53 ^f^	16.38 ± 0.28 ^ac^	14.76 ± 1.8 ^adg^	69.51 ± 1.13 ^b^	108.71 ± 5.49 ^e^
KA	39.19 ± 0.14 ^c^	127.25 ± 6.00 ^e^	13.14 ± 0.12 ^d^	14.45 ± 0.97 ^adg^	34.40 ± 0.56 ^c^	51.93 ± 4.58 ^d^
KG	31.40 ± 1.25 ^d^	111.40 ± 5.00 ^g^	11.00 ± 0.34 ^e^	12.95 ± 1.09 ^dg^	34.56 ± 0.73 ^c^	48.43 ± 4.82 ^d^
**Cones**						
RA	71.72 ± 1.31 ^a^	102.28 ± 8.60 ^e^	18.64 ± 0.40 ^a^	17.88 ± 1.87 ^abcf^	65.14 ± 0.78 ^a^	92.82 ± 4.8 ^bf^
FT	78.18 ± 0.65 ^b^	122.11 ± 5.50 ^f^	17.87 ± 0.38 ^b^	16.50 ± 1.62 ^acdfg^	87.16 ± 0.82 ^b^	101.97 ± 10.02 ^f^
FD	72.39 ± 0.30 ^c^	134.77 ± 12.87 ^f^	19.19 ± 0.46 ^ac^	12.65 ± 2.21 ^dg^	74.29 ± 0.79 ^c^	95.68 ± 9.27 ^bdf^
KA	76.91 ± 2.43 ^d^	91.74 ± 8.71 ^e^	14.45 ± 0.43 ^d^	12.66 ± 1.68 ^cdg^	82.42 ± 1.28 ^d^	69.34 ± 5.14 ^aceg^
KG	76.73 ± 1.67 ^d^	120.82 ± 7.69 ^ef^	23.31 ± 0.58 ^e^	20.18 ± 0.61 ^cf^	70.80 ± 0.93 ^e^	66.63 ± 5.53 ^aceg^

Sample codes see in Table 4. Results represent the average value of three repetitions^. a–g^—different letters indicate significant differences among varieties or methods (*p* ≤ 0.05).

**Table 2:** Main volatile compounds and their percentage composition in the supercritical fluid extract of leaves and cones of analyzed Lithuanian hop varieties (Rt—average retention time of compound, min; KI—Kovats index; sample codes see in Table 4; RSD ≤ 5.8%, *n* = 3).

R_t_, min	Identified Compound	KI	Leaves					Cones				
			RA	FT	FD	KA	KG	RA	FT	FD	KA	KG
7.277	β-Pinene	983						0.41	0.43	0.51	0.52	0.42
7.853	β-Myrcene	995						26.88	26.49	23.42	32.62	34.59
9.439	Sylvestrene	1028						0.17	0.16	0.20	0.26	0.32
16.737	Borneol	1166	0.52	3.55	1.91	1.76	1.68					
18.898	Estragole	1203	1.82	12.17	5.20	7.31	17.94	0.12				
29.074	Ylangene	1368						0.08	0.10	0.10	0.06	0.06
29.349	Copaene	1373		0.89	0.94	1.22		0.32	0.43	0.42	0.22	0.22
31.923	β-Caryophyllene	1415	2.48	13.92	24.81	16.85	13.59	9.65	12.14	12.56	7.62	7.84
32.550	β-Cubebene	1426			0.94			0.42	0.54	0.53	0.33	0.36
33.111	α-Bergamotene	1435						0.93	0.72	0.70	0.88	0.85
33.984	α-Humulene	1449	8.55	38.01	50.56	58.55	50.06	34.49	37.09	37.31	27.59	27.79
34.586	β-Farnesene	1460	0.96	3.01	4.17	3.77	5.02	21.24	15.21	14.59	23.21	20.57
35.279	β-Cadinene	1471						0.05	0.22	0.22	0.15	0.12
35.496	γ-Muurolene	1475	0.23	1.43	2.66	1.50	1.99	0.60	0.90	0.91	0.63	0.64
35.936	β-Selinene	1482						0.24	0.34	0.37	0.39	0.35
36.541	γ-Amorphene	1492			0.79			0.36	0.55	0.60	0.43	0.36
36.903	α-Farnesene	1498						0.53	0.88	1.06	0.86	0.26
37.662	γ-Cadinene	1512	0.74	1.67	2.15	2.97	2.53	1.11	1.29	1.42	1.25	0.92
38.292	δ-Cadinene	1523	1.01	2.91	3.77	4.77	3.25	1.41	1.73	1.99	1.33	1.19
38.722	*trans*-Cadina-1,4-diene	1530						0.13	0.13	0.18	0.15	0.11
39.030	α-Muurolene	1536						0.11	0.13	0.17	0.08	0.11
41.494	Caryophyllene oxide	1579						0.15	0.05	0.07	0.10	0.23
42.996	Humulene epoxide II	1606				1.30	3.14	0.44	0.19	0.22	0.41	0.87
-	Sum of minor compounds ^a^		83.69	22.44	2.10	0.00	0.80	0.16	0.28	2.45	0.91	1.82

^a^ Compounds which were identified in less than four samples: 2-methylpropyl 2-methylpropanoate (KI 941), decane (KI 1001), 2-mehylbuthyl 2-mehylpropanoate (KI 1018), β ocimene (KI 1048), linalool oxide (KI 1073), (3 methyl 1-(3-methyl-2-furyl)-1-butanone (KI 1206), 2-undecanone (KI 1299), 2,5-bornanedione (KI 1304), naginata ketone (KI 1306), methyl 4-decenoate (KI 1314), 3,3,6 trimethyl-1,5 heptadien-4-one (KI 1318), unidentified compound (1) (KI 1320), methyl nerolate (KI 1329), α-cubebene (KI 1348), β-bourbonene (KI 1381), α-amorphene (KI 1479), geranyl isobutyrate (KI 1518), α calacorene (KI 1541), and unidentified compound (2) (KI 1595).

**Table 3:** Xanthohumol content (% of dry weight) in leaves and cones of Lithuanian hop varieties (RSD ≤ 3.8%, *n* = 3).

Variety	Xanthohumol Content (% of Dry Weight)	
	Leaves	Cones
‘Raudoniai’	0.0014 ^a^	0.1466 ^f^
‘Fredos taurieji’	0.0080 ^b^	0.1181 ^g^
‘Fredos derlingieji’	0.0067 ^c^	0.1295 ^h^
‘Kauno ankstyvieji’	0.0059 ^d^	0.1413 ^f^
‘Kauno gražieji’	0.0030 ^e^	0.2136 ^i^

^a–i^—different letters indicate significant differences among varieties (*p* ≤ 0.05).

**Table 4:** Hop varieties analyzed in this study.

Variety	Sample Code	Country of Origin	Voucher No.
‘Raudoniai’	RA	Lithuania	V00015
‘Fredos taurieji’	FT	Lithuania	V00012
‘Fredos derlingieji’	FD	Lithuania	V00011
‘Kauno ankstyvieji’	KA	Lithuania	V00014
‘Kauno gražieji’	KG	Lithuania	V00013

## Data Availability

Data available from the authors.

## Sample Availability

Samples of the compounds are not available from the authors.
